# Case Report: Unilateral resistance and impact loading during knee rehabilitation after grade-2 MCL injury was associated with hip-specific aBMD accrual in an elite female road cyclist

**DOI:** 10.3389/fspor.2026.1823271

**Published:** 2026-04-23

**Authors:** Stefan Pettersson, Lykke Tamm, Angelica Lindén Hirschberg, Jonatan Fridolfsson

**Affiliations:** 1 Center for Health and Performance, Department of Food and Nutrition and Sport Science, University of Gothenburg, Gothenburg, Sweden; 2 Swedish Olympic Committee, Sofiatornet, Olympiastadion, Stockholm, Sweden; 3 Department of Women's and Children's Health, Karolinska Institutet and Department of Gynecology and Reproductive Medicine, Karolinska University Hospital, Stockholm, Sweden; 4 Center for Lifestyle Intervention, Institute of Medicine, University of Gothenburg, Gothenburg, Sweden; 5 Department of Medicine, Geriatrics and Emergency Medicine Östra, Sahlgrenska University Hospital, Gothenburg, Sweden

**Keywords:** bone remodeling, case report, DXA, endurance athlete, lean mass, mechanical loading, osteogenic loading, relative energy availability

## Abstract

**Background:**

Low areal bone mineral density (aBMD) is prevalent in elite road cyclists due to non-weight-bearing training loads and, at times, low energy availability (EA). Real-world, site-specific skeletal responses to targeted loading during rehabilitation remain insufficiently characterized.

**Case presentation:**

A 24-year-old WorldTour-level female road cyclist with low aBMD (Z-score ≤ −1.1) initiated a periodized resistance and impact loading program and daily vitamin D (800 IU). Three fasted-morning DXA and venous blood assessments (Oct-2024; Mar-2025; May-2025) were paired with training logs, dual-sided crank data, menstrual tracking, and weighed diet records. Five days after second DXA, a left grade-2 medial collateral ligament knee sprain precluded loading of the injured limb for ∼11 weeks; rehabilitation emphasized heavy unilateral resistance work of the contralateral limb while cycling volume was maintained.

**Results:**

From baseline to pre-injury, lumbar and bilateral femoral aBMD increased (all ≥least significant change, LSC). During unilateral rehabilitation, only the trained femur accrued further aBMD (total hip +2.8%; neck +3.8%; both ≥LSC), whereas the injured femur was stable/slightly lower (≤LSC). Whole-body BMD rose further; and leg-specific lean mass diverged (+uninjured/−injured). Right-leg pedal dominance (∼52%–54%) was similar in the 2024 and 2025 seasons, making cycling-related asymmetry an unlikely explanation for the March–May divergence. Across the three sampling time points, cortisol, 25-hydroxyvitamin D, thyroid hormones, and sex hormones were within reference ranges. Menstrual bleeding reappeared in late November 2024 but remained irregular; EA before baseline was frequently low, and after baseline it was assessed once and appeared higher, but no temporal trend can be inferred.

**Conclusion:**

Progressive resistance and impact loading were associated with rapid bilateral aBMD accrual pre-injury, and unilateral high-strain loading during knee rehabilitation after a grade-2 MCL sprain coincided with site-specific increases in femoral aBMD despite preserved cycling volume. These findings highlight rehabilitation periods as practical windows to deliver targeted bone-loading in non-weight-bearing endurance athletes and support the feasibility of brief, targeted bone-loading as an in-season maintenance strategy, while contributions from energetic and reproductive factors cannot be excluded.

## 1 Introduction

Low areal bone mineral density (aBMD) is common in elite road cyclists, reflecting the sport's non-weight-bearing nature, high training volumes, and in some cases, low energy availability (EA). These factors contribute to impaired bone health and increased fracture risk, with a substantial proportion of professionals presenting lumbar spine or hip Z-scores below −1.0 and some meeting osteoporosis criteria (1, 2). For athletes, sport-specific risk frameworks emphasize more stringent thresholds than in the general population (3–5).

To address these deficits, resistance and impact-loading are recommended countermeasures in low-impact endurance sports, yet their site-specific effects under ecologically valid, elite performance conditions remain incompletely characterized (6, 7). Cross-sectional evidence suggests that cyclists who perform resistance training exhibit higher hip and lumbar BMD than those who do not (8), while short bouts of high-impact exercise (e.g., jumping, bounding) may offer potent osteogenic stimuli without excessive interference with endurance training adaptations (6, 7). Moreover, periods of asymmetric mechanical loading, resulting from habitual side preference or injury, provide natural opportunities to observe localized skeletal adaptation (9, 10). Beyond densitometric indices, recent clinical data indicate that Relative Energy Deficiency in Sport (REDs) is characterized by a catabolic bone phenotype, with suppressed bone formation and elevated resorption, which compromises mechano-adaptation and lowers BMD, particularly at weight-bearing sites, and is associated with higher stress-fracture rates (11).

Against this background, the present case offered a unique opportunity to examine these principles *in vivo*. Here we report a 24-year-old professional female road cyclist in whom a traumatic left medial collateral ligament (MCL) knee sprain, together with a standardized resistance/impact-loading program and maintained cycling volume, provided a period of unilateral heavy loading. Over seven months, we tracked skeletal, body composition, and blood biomarker outcomes at three standardized fasted morning DXA assessments and retrospectively contextualized these changes with training load, pedal power asymmetry, EA, and menstrual function, while menstrual bleedings and serial skinfolds were routinely recorded during the 12 months preceding baseline. Our objective was to characterize the osteogenic response to targeted unilateral mechanical strain during rehabilitation in a WorldTour-level athlete. We hypothesized that the limb receiving targeted high-strain loading would exhibit site-specific femoral aBMD gains exceeding the least significant change (LSC), while the contralateral injured limb—exposed only to cycling and daily activities—would not.

## 2 Case description

The participant was a 24-year-old professional female road cyclist competing at the Olympic Games, UCI Road World Championships, European Championships, and multiple Women's WorldTour races. Menarche occurred at age 12; she reported a 4–5-month episode of secondary amenorrhea at 15–16 years and ∼1 year of secondary amenorrhea from late 2023, with menses resuming on 25 November 2024 No relevant family, psychosocial, or genetic history affecting bone health was reported.

On 30 October 2024 she underwent a fasted morning baseline assessment of aBMD, body composition, and blood biomarkers between 08:00 and 09:00. Dual-energy x-ray absorptiometry (DXA; GE Lunar iDXA, GE Healthcare, Madison, WI, USA) assessed aBMD at the lumbar spine, total femur, and femoral neck, as well as whole-body BMD and bone mineral content (BMC), lean mass (LM), and fat mass (FM). Blood samples obtained at each visit were analyzed at accredited clinical laboratories, in accordance with the International Standard ISO 15189:2022, using standardized and quality-controlled clinical chemistry methods for serum cortisol, 25-hydroxyvitamin D, thyroid hormones, gonadotropins and sex hormones. Following notable post-injury DXA findings, all ancillary data were compiled retrospectively: training volume and body mass from TrainingPeaks® (including pedal asymmetry and cycling exercise energy expenditure estimated via a dual-sided crank power meter; Shimano FC-R9200-P), dietary intake from weighed food diaries (*n* = 61 days in total) logged in MyFitnessPal®, menstrual bleeding records, and serial seven-site skinfolds (*Σ*7SF; measured by a sports dietitian certified as an ISAK Level II anthropometrist) to estimate fat-free mass (FFM) and compute EA (see Supplementary Methods). The participant provided written informed consent for in-depth analysis.

### 2.1 Baseline measurements and interventions

**Figure 1:**
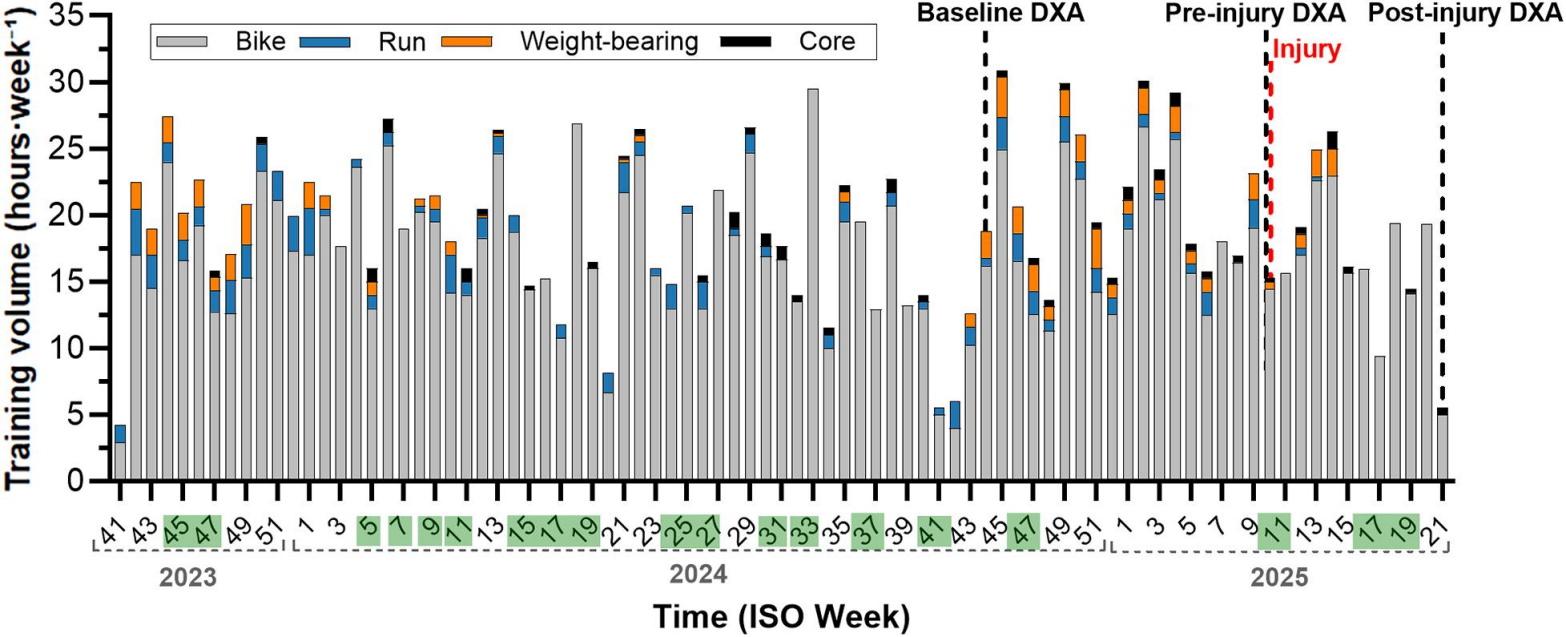
Weekly training volume (hours·week⁻^1^) by modality from 2023 to the post-injury DXA (week 21, 2025). Vertical dashed lines indicate DXA assessments; the red dashed line marks the knee injury (8 Mar 2025). Green-shaded weeks denote weeks including competition day**(s)**. Weight-bearing denotes resistance and impact-loading training.

At baseline, aBMD Z-scores were low at clinically relevant sites (Table 1). As shown in Figure 1, the athlete had not engaged in systematic resistance training between February and October 2024. A structured program was therefore initiated in November 2024 and implemented in three progressive phases, performed two to three times per week. The adaptation phase (November; 3–4 sets ×  8–12 repetitions at moderate loads) introduced bilateral and unilateral multi-joint lower-limb exercises including single-leg squat, trapbar deadlift, split squat, Romanian deadlift, squat, and leg press, each preceded by 10 min of targeted rehabilitation (hip abduction, glute activation) and followed by 10 min of core and mobility work. The strength phase (December–January) progressed to heavy loads (4–5 sets × 5–6 repetitions). The power phase (February–March) returned to moderate loads (3–4 sets × 5–6 repetitions) and added plyometric impact drills (box jumps, skater jumps, jumping lunges, mountain climbers). After the MCL injury on 8 March, the programme was modified: plyometrics and single-leg work with the injured limb were excluded; bilateral lifts were modified to emphasize the uninjured side; and each session began with 5–10 min of VMO and upper-limb prehab. Impact exercises (box jumps, weighted vertical jumps, mountain climbers) were maintained for the uninjured side. Full session-by-session details are provided in the Supplementary material. On medical advice, vitamin D supplementation (800 IU·day⁻^1^) was started in November 2024 and maintained thereafter. A follow-up DXA was scheduled four months later. During the intervention, the participant received individualized dietary counselling and adapted her peri-exercise—particularly in-ride—energy intake during cycling sessions.

**Table 1:** DXA outcomes (Z-scores) and blood biomarkers at each time point; laboratory reference intervals shown for context.

Outcome measure	Baseline (Oct-24)	Pre-injury (Mar-25)	Post-injury (May-25)	Absolute BMD Baseline g/cm^2^	Change Oct-Mar (%)	Change Mar-May (%)	Change Oct-May (%)
**aBMD (Z-score** ^a^ **)**				1.049			
AP spine (L1-L4)	−1.1	−0.9	−0.8	0.832	2.3	1.8	4.1
Left Femur (total)	−1.4	−0.9	−0.9	0.870	6.7	−0.1	6.6
Right Femur (total)	−1.1	−0.8	−0.6	0.780	4.4	2.8	7.2
Left Femur (neck)	−1.9	−1.6	−1.6	0.815	5.3	−0.7	4.5
Right Femur (neck)	−1.6	−1.5	−1.2	1.049	2.3	3.8	6.3
**Blood marker**							
Total testosterone (nmol/L)	0.7	0.8	0.17	0.3–2.5			-
Estradiol (pmol/L)	884	398	178	70–530			
Progesterone (nmol/L)	0.9	1.3	0.47	0.3–4.5			
FSH (IU/L)	3.6	5.4	5.33	3–8			
LH (IU/L)	6.1	5.4	4.51	2–12			
Cortisol (nmol/L)	215	386	473.1	170–540			
TSH (mIU/L)	3	1.7	1.76	0.4–4.0			
Free T3 (pmol/L)	7.1	5.3	5	3.5–6.5			
25-hydroxyvitamin D (nmol/L)	99	91	100.3	≥ 50			

Z-scores are shown for reference only and were not used in these calculations. Z-scores are based on manufacturer-derived, site-specific reference data. aBMD, areal bone mineral density; FSH, Follicle-stimulating hormone; LH, Luteinizing hormone; TSH, Thyroid-stimulating hormone; Free T3, Free triiodothyronine.

a Percent change is calculated from absolute BMD values (g·cm⁻^2^; see Figure 3A).

### 2.2 DXA precision and least significant change (LSC)

Precision error values used to interpret change were taken from studies using the same Lunar iDXA model. For normal-weight adult women (*n* = 34), least significant change (LSC) values were 0.014 g·cm⁻^2^ for lumbar spine (L1–L4), 0.017 g·cm⁻^2^ for total hip, and 0.025 g·cm⁻^2^ for femoral neck (12). For whole-body outcomes in elite endurance and power athletes on the same device, LSCs were 0.016 g·cm⁻^2^ for whole-body BMD, 21 g for whole-body BMC, and 417 g for leg LM (13). Observed changes are therefore reported as ≥LSC (exceeding measurement variability) or <LSC. To aid interpretation, observed BMD changes are also expressed as multiples of site-specific precision error (PE = LSC/2.77), providing a standardized metric for the magnitude of change relative to measurement variability (14).

## 3 Results

### 3.1 Training load, injury, and rehabilitation

Weekly training volume by modality is shown in Figure 1. With a specific focus on osteogenic activity, weight-bearing training (resistance + impact-loading) was concentrated in the off-season, averaging 1.3 and 1.6 h·week⁻^1^ in 2023–2024 and 2024–2025 (weeks 42–4 and 41–6), respectively; during the 2024 competition period (weeks 5–40), strength training averaged 0.14 h·week⁻^1^. DXA was performed at baseline (30 October 2024), pre-injury (3 March 2025), and post-injury (21 May 2025). On 8 March 2025, five days after the second DXA, the athlete sustained a traumatic left-knee injury (MRI: grade-2 MCL sprain). During the subsequent 11-week rehabilitation period, plyometrics and single-leg work with the injured limb were excluded; bilateral lifts were modified to emphasize the uninjured side (Rehabilitation program in Supplementary material).

### 3.2 Baseline to Pre-injury

**Figure 2:**
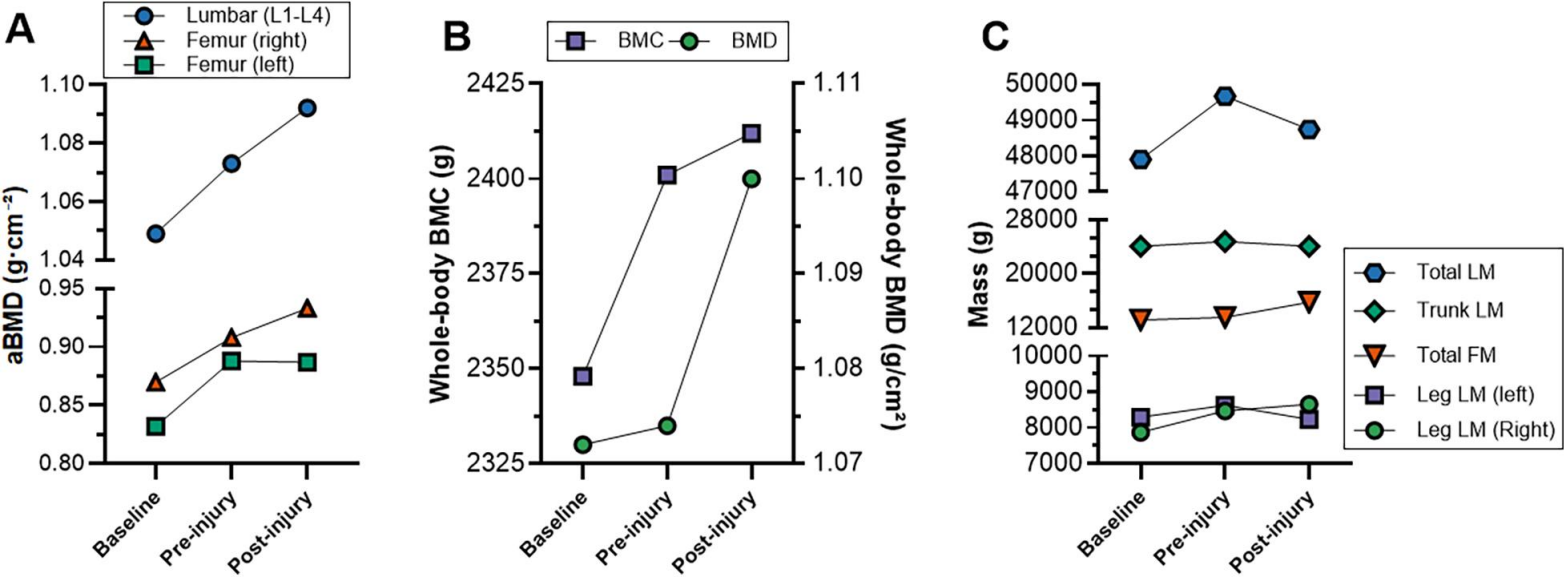
Changes in **(A)** lumbar spine and femoral aBMD, **(B)** whole-body BMC and BMD, and **(C)** lean and fat mass distribution across baseline, pre-injury, and post-injury assessments in an elite female road cyclist. aBMD, areal bone mineral density; BMC, Bone mineral content; LM, Lean mass; FM, Fat mass.

The introduction of structured resistance and impact loading was followed by bilateral gains in aBMD (Figure 2A; Table 1). Lumbar spine aBMD increased by 2.3% (≥LSC). Total hip and femoral neck aBMD increased in both limbs (right: +4.4% and +2.3%; left: +6.6% and +5.3%), with all changes ≥LSC. Whole-body BMD and BMC increased by 1.9% and 0.7%, respectively (≥LSC), while leg LM rose by 2.5% (>LSC) (Figures 2B,C).

### 3.3 Pre-injury to post-injury

Adaptations during rehabilitation were side-specific (Figure 2; Table 1). In the uninjured (right) limb, total hip aBMD increased by 2.8% (+0.025 g/cm^2^, 4.1 × PE, ≥LSC), whereas the injured (left) limb remained unchanged (−0.1%, −0.001 g/cm^2^, −0.1 × PE). At the femoral neck, the trained side gained 3.8% (+0.032 g/cm^2^, 3.5 × PE, ≥LSC), while the injured side declined slightly (−0.7%, −0.006 g/cm^2^, −0.6 × PE). LM increased in the uninjured leg (+2.2%) but decreased in the injured leg (−4.6%); total LM declined (−1.9%), primarily due to reduced trunk LM (−2.9%), while total FM increased (+16.5%) (Figure 2C). Lumbar spine aBMD increased by 1.8% (3.8 × PE, ≥LSC), while whole-body aBMD and BMC rose by 2.4% (≥LSC) and 0.5% (<LSC), respectively. Over the full seven-month observation period, all site-specific aBMD changes exceeded LSC with PE ratios ranging from 3.9 to 10.4.

### 3.4 Pedal asymmetry, energy availability, menstrual function, and biomarkers

**Figure 3:**
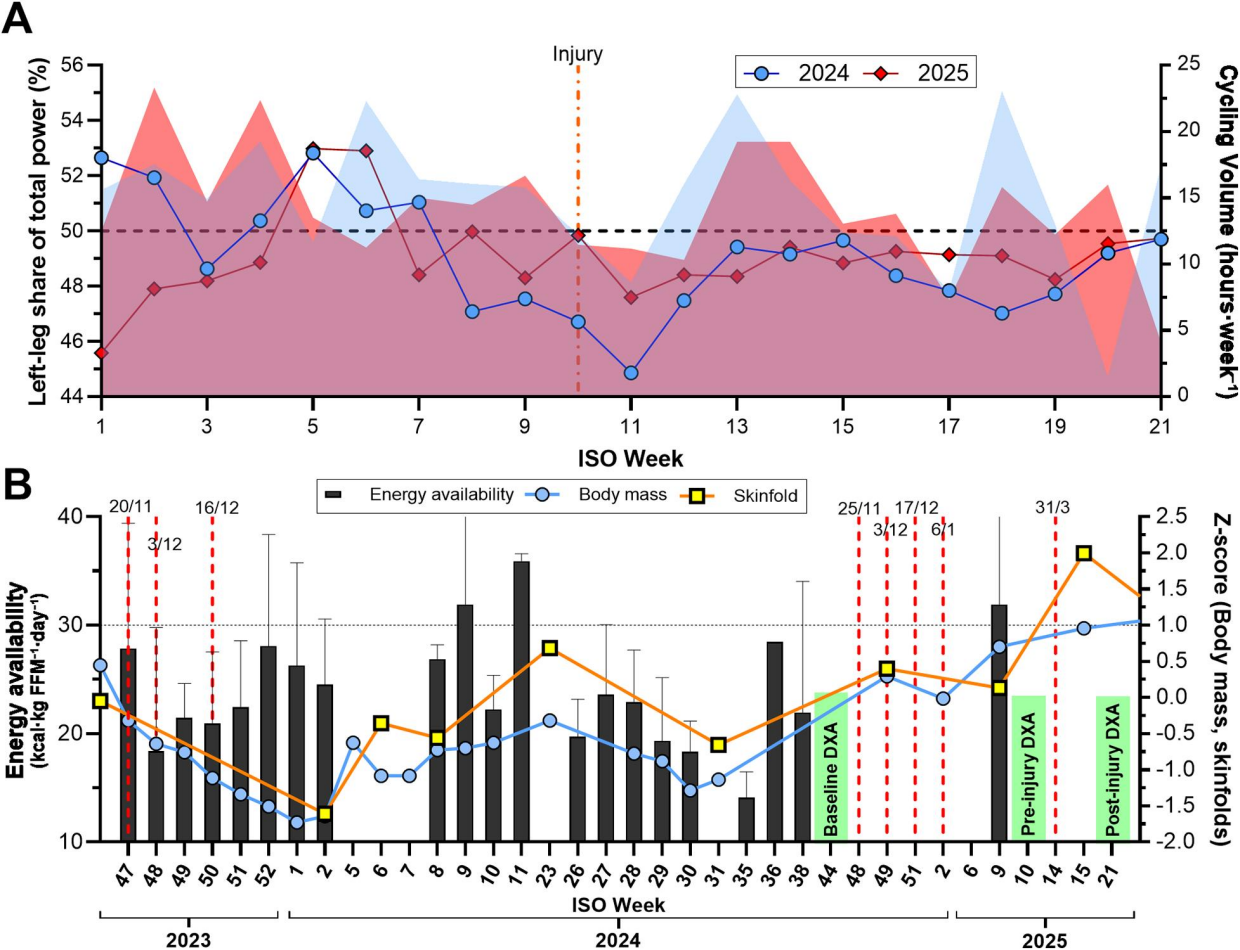
**(A)** weekly left–right power balance and cycling volume across weeks 1–21 of 2024 (pre-injury) and 2025 (injury and rehabilitation). **(B)** Energy availability and standardized body mass and skinfold thickness (Z-scores) from late 2023 through May 2025. Red dashed lines indicate menstrual bleeding onsets; green labels denote DXA assessments. Panel **(A)** Left/right balance is the percentage of total power from the left leg (50% = symmetry) and shaded areas represent total cycling volume (hours·week⁻^1^); **(B)** The horizontal dashed line indicates the EA threshold (30 kcal·kg FFM⁻^1^·day⁻^1^). Calendar dates (dd/mm) are displayed at bleeding onsets.

Retrospective dual-sided crank data showed a stable right-leg dominance (52%–54% of total power) across weeks 1–21 of 2024 (pre-injury) and 2025 (injury and rehabilitation period), despite similar weekly cycling volume (Figure 3A).

Figure 3B summarizes the year preceding the baseline DXA and the subsequent observation/intervention period through May 2025 (including the injury and rehabilitation). Secondary amenorrhea persisted for 345 days (16 December 2023–25 November 2024). During this interval, EA was frequently below the operational threshold (30 kcal·kg FFM⁻^1^·day⁻^1^). In the post-baseline period, EA was assessed once and was higher than earlier values, but a trend cannot be inferred from a single time point. Menses reappeared on 25 November 2024 but remained irregular. Across the three sampling time points, all measured blood biomarkers (including cortisol, 25-hydroxyvitamin D, and sex hormones) were within laboratory reference intervals (Table 1), and the participant was anovulatory at each sampling occasion.

## 4 Discussion

Elite road cyclists face a distinct skeletal risk profile, combining near-complete absence of weight-bearing stimuli with high training volumes and, at times, low EA, with potential long-term health consequences beyond the competitive career. The present case offered a unique opportunity to examine site-specific osteogenic responses to targeted loading under real-world elite conditions, and to explore rehabilitation as a practical bone-loading window in a non-weight-bearing sport. In the present case, a structured program of resistance and impact loading was followed by bilateral gains at the lumbar spine and femur from October 2024 to March 2025 (≥LSC). After a left MCL knee sprain, only the deliberately loaded right limb continued to accrue femoral aBMD over ∼11 weeks, while the injured left limb remained unchanged or slightly lower despite maintained cycling volume and stable right-leg pedal dominance across comparable seasons; leg-specific LM changed in the same direction. Taken together, the temporospatial pattern is most consistent with load-driven, site-specific adaptation in the trained limb, in line with mechanostat expectations (15, 16).

EA and menstrual function can modulate osteogenic responsiveness in athletes (11, 17–21). In this case, EA during the amenorrheic interval before baseline was frequently <30 kcal·kg FFM⁻1·day⁻1, consistent with dose–response suppression of bone turnover at low EA (19) and the skeletal risk profile described in REDs (11). Menstrual bleedings reappeared in late 2024 but they remained irregular until the end of this case study in May 2025. In this case, luteinizing hormone and free triiodothyronine were within reference ranges at all sampling points and therefore appeared unaffected, which should be noted. Classically, resumption of menses has been associated with spinal aBMD gains in athletes (20), and experimental work indicates that EA influences luteinizing hormone pulsatility and triiodothyronine economy (17, 18, 21). Collectively, the temporal and site-specific patterns observed are consistent with a renewed osteogenic responsiveness to mechanical loading; a contributory role of improvements in the energetic/reproductive milieu cannot be excluded, but post-baseline EA was reassessed only once and menstrual function remained irregular, limiting inferences about systemic recovery.

Before March 2025, the bilateral increases likely reflected the combination of novel osteogenic stimuli (resistance/impact) and a more favorable energetic context relative to the pre-baseline period. Thereafter, the unilateral rehabilitation phase provided a quasi-natural experiment: only the trained femur accrued further density while endocrine markers and vitamin D status remained within reference ranges. Retrospective crank-based data documented stable right-leg dominance (∼52%–54%) across comparable seasons, making cycling asymmetry an unlikely explanation for the March–May divergence. This pattern accords with mechanostat principles that predict local adaptation to strain magnitude and rate (15, 16). Heavy unilateral lifts and impact drills likely provided the necessary high-strain, high-rate stimuli absent in cycling alone (6, 7). This localized response suggests that targeted resistance may contribute to offsetting sport-specific skeletal deficits. From a maintenance perspective, the athlete's in-season exposure to weight-bearing exercise (∼0.14 h·week^⁻1^) was likely below the minimal effective dose to sustain BMD. Randomized data in elite road cyclists show that brief, frequent impact “micro-doses” (e.g., five 5-min jump bouts per week) can preserve femoral-neck BMD over 18 weeks off-season (22). Beyond impact micro-dosing, twice-weekly high-intensity resistance plus impact (∼30 min·session^⁻1^) improves spine and hip BMD in adults with low bone mass (23), and either 2 sessions·week^⁻1^ of resistance training or 3 sessions·week^⁻1^ of jump training over 12 months increases BMD in osteopenic men (24). Taken together, these trials suggest—and the present case provides supporting observational context—that one to two short sessions·week^⁻1^, or distributed daily impact, constitute a pragmatic minimal dose for maintenance. Observational evidence that elite cyclists often have low BMD despite heavy lower-limb resistance training underscores the importance of targeted impact loading (25), while season-long declines in bone markers without dedicated bone loading further argue for an in-season maintenance dose (26). In this case, targeted unilateral high-strain loading coincided with femoral aBMD gains beyond LSC over ∼11 weeks despite preserved cycling volume, providing prospective within-athlete evidence consistent with a minimal in-season bone-loading dose. To contextualize the clinical magnitude of the observed changes, a meta-regression found that a 2% improvement in total hip BMD was associated with approximately 28% and 16% reductions in vertebral and hip fracture risk, respectively (27). Although derived from pharmacological trials in postmenopausal women and not directly extrapolatable to a young premenopausal athlete, these benchmarks suggest that the 7.2% total hip BMD gain observed in the trained limb over seven months (+0.064 g/cm^2^, 10.4 × PE) could carry meaningful implications for long-term skeletal health in this at-risk population, if sustained.

### 4.1 Strengths and limitations

The validity of these findings is supported by several methodological strengths: All three DXA assessments were performed by the same technologist on the same iDXA system and analysis platform as in Pettersson et al. (13). Whole-body LSC values were anchored to ISCD recommendations (14) and were comparable or lower than published precision data (12). For hip and lumbar spine aBMD, published *in-vivo* iDXA coefficients (12) were applied as conservative LSC thresholds. The observed unilateral femoral gains clearly exceeded these limits, indicating true biological adaptation rather than measurement variability. Other strengths include objective cycling asymmetry data, and retrospectively recorded training, menstrual, and nutrition logs. Several limitations must be acknowledged. As an inherent property of the single-case design (*n* = 1), no statistical inference or population-level generalisation is possible, and the findings are strictly hypothesis-generating. Factors unique to this individual may have modulated the skeletal response in ways that would not replicate across a cohort, and controlled trials are required to test whether the observed associations reflect causal mechanisms. The reliance on aBMD is a further constraint, as it cannot distinguish geometric from material adaptations. Furthermore, although exercise type, frequency, and set-rep progression are reported, the absence of documented load progression in the resistance training programme precludes quantification of the mechanical stimulus, limiting the precision with which the osteogenic dose can be characterized. EA was assessed at a single post-baseline time point, consistent with methodological practice in the broader literature on low EA in elite athletes, where single-timepoint dietary assessments have similarly been employed (28). Indirect indicators of cumulative low EA exposure, including menstrual history, BMD trajectory, and metabolic hormone concentrations, may serve as more robust proxies for chronic energy status and were all documented in the present case. Post-baseline dietary counselling focused on increasing overall energy intake, but repeated quantitative EA assessments were not implemented, precluding characterisation of the energetic milieu during the intervention and rehabilitation periods. Given the well-established role of EA in modulating bone metabolism and mechano-responsiveness (17–19), this represents a major limitation: whether improvements in EA contributed to the observed aBMD gains, independently of or synergistically with mechanical loading, cannot be determined. Persistent menstrual irregularity through May 2025 further constrains inferences about systemic reproductive recovery.These factors collectively temper generalizability and motivate future longitudinal work with repeated EA assessments, continuous monitoring of ovulatory status, and evaluation of bone microarchitecture.

### 4.2 Practical implications for rehabilitation and return-to-sport

Based on the association observed in this case, periods of enforced unilateral unloading during knee rehabilitation may represent an underused opportunity to deliver safe, structured contralateral osteogenic loading without materially reducing cycling volume. Based on the present observations, we suggest the following practical considerations for elite road cyclists with low aBMD:

• Use rehabilitation as an opportunity: maintain contralateral heavy loading when the injured limb cannot tolerate high strain or impact.
• Prioritise hip-directed stimuli: combine heavy unilateral lifts with brief, well-tolerated impact drills when clinically appropriate to restore weight-bearing exposure absent in cycling.
• Aim for a minimal maintenance dose: one to two short sessions per week (or brief distributed impact bouts) may be a pragmatic target during periods with low weight-bearing exposure.
• Interpret adaptations in context: track limb-specific lean mass and, where available, crank-based asymmetry, while considering energy availability and menstrual function as modifiers of mechano-responsiveness.

## 5 Conclusion

In a professional road cyclist with low baseline aBMD, progressive resistance and impact loading were associated with bilateral aBMD gains pre-injury, and unilateral high-strain loading during rehabilitation coincided with site-specific femoral accrual beyond LSC despite preserved cycling volume. While contributions from EA and reproductive status cannot be excluded, as menses remained irregular through May 2025 and post-baseline EA was reassessed once, the temporospatial pattern is most consistent with load-driven adaptation. Given the single-case design, these observations are hypothesis-generating and not generalizable; causal inference is not possible. These findings underscore the central role of mechanical loading and motivate prospective trials to define a minimal effective in-season bone-loading dose, alongside serial assessments of EA, ovulatory status, and bone microarchitecture.

## 6 Patient perspective

In the weeks preceding the baseline DXA, the athlete had planned to increase resistance training during the off-season to enhance performance, citing perceived weakness during all-out efforts. The unexpectedly low BMD findings were surprising and stressful but ultimately strengthened adherence to the resistance program and prompted a further increase in energy intake. The athlete reported a reframing of priorities: reducing the emphasis on weight loss and aiming to maintain structured resistance training during the competitive period to the extent feasible within a schedule of approximately 40–45 races per season.

## Data Availability

The original contributions presented in the study are included in the article/Supplementary Material, further inquiries can be directed to the corresponding author.
