# Supplementary material for: Case Report: Unilateral resistance and impact loading during knee rehabilitation after grade-2 MCL injury was associated with hip-specific aBMD accrual in an elite female road cyclist
Source: Front Sports Act Living. 2026 Apr 23;8:1823271. doi: 10.3389/fspor.2026.1823271 (PMC13149270; doi:10.3389/fspor.2026.1823271)
Supplement: Supplementary file 1 [file Datasheet1.pdf]

Supplementary Methods: Energy availability

Energy availability (EA). Daily EA was computed as dietary energy intake (EI) minus *net* exercise energy expenditure (EEE_{net}), normalized to fat-free mass (FFM):

$$\mathrm{EA}\ (\mathrm{kcal}\cdot\mathrm{kg\,FFM}^{-1}\cdot\mathrm{day}^{-1}) = \frac{\mathrm{EI}\ (\mathrm{kcal}\cdot\mathrm{day}^{-1}) - \mathrm{EEE}_{\mathrm{net}}\ (\mathrm{kcal}\cdot\mathrm{day}^{-1})}{\mathrm{FFM}\ (\mathrm{kg})}.$$

EEE_{net} was defined as gross exercise energy cost minus the resting metabolic energy that would have occurred during the exercise time:

$$\mathrm{EEE}_{\mathrm{net}} = \mathrm{EEE}_{\mathrm{gross}} - \left(\frac{\mathrm{RMR}}{24} \times t_{\mathrm{ex}}\ [\mathrm{h}]\right).$$

FFM came from skinfold calculations; EI was taken from weighed food records.

Resting metabolic rate (RMR). RMR was estimated from FFM using the Cunningham equation:

$$RMR\ (kcal \cdot day\text{^}\{-1\}) = 500 + 22 \times FFM\ (kg)$$

Reference:

Cunningham JJ. *A reanalysis of the factors influencing basal metabolic rate in normal adults.* **Am J Clin Nutr.** 1980;33(11):2372–2374.
