# Supplementary material for: Case Report: Unilateral resistance and impact loading during knee rehabilitation after grade-2 MCL injury was associated with hip-specific aBMD accrual in an elite female road cyclist
Source: Front Sports Act Living. 2026 Apr 23;8:1823271. doi: 10.3389/fspor.2026.1823271 (PMC13149270; doi:10.3389/fspor.2026.1823271)
Supplement: Supplementary file 2 [file Table1.docx]

| Rehab |  | 10min |  |
| --- | --- | --- | --- |
| Exercise | Sets | Reps | Weight/Equipment |
| Crab walk | 3 | 12 | Band |
| SL bridge elevated | 3 | 12 | Body weight/light |
| SL squat | 4 | 8 | Moderate |
| Trapbar deadlift | 4 | 8 | Moderate |
| Bird dog | 3 | 12 | Body weight |
| Core and mobility |  | 10 min |  |

**Adaptation Session One**

**Adaptation Session Two**

| Rehab |  | 10 min |  |
| --- | --- | --- | --- |
| Exercise | Sets | Reps | Weight/Equipment |
| 4 point kneeling banded hip abduction | 3 | 12 | Band |
| Step up | 3 | 12 | Light |
| Single RDL | 4 | 8 | Moderate |
| Squat | 4 | 8 | Moderate |
| Glute kick backs | 3 | 12 | Body weight/band |
| Core and mobility |  | 10 min |  |

**Adaptation Session Three**

| Rehab |  | 10 min |  |
| --- | --- | --- | --- |
| Exercise | Sets | Reps | Weight/Equipment |
| Swiss ball hamstring bridge holds | 4 | 10 sec | Swiss ball |
| Bridge with hip external rotation | 3 | 12 | Band |
| Split squat | 4 | 8 | Moderate |
| Leg press | 4 | 8 | Moderate |
| Forearm side plank with hip abduction | 3 | 12 | Body weight |
| Core and mobility |  | 10 min |  |

**Strength Session One**

| Rehab |  | 10 min |  |
| --- | --- | --- | --- |
| Exercise | Sets | Reps | Weight/Equipment |
| Crab walk | 3 | 15 | Band |
| SL bridge elevated | 4 | 6 | Moderate |
| SL squat | 5 | 5 | Heavy |
| Trapbar deadlift | 5 | 5 | Heavy |
| Core and mobility |  | 10 min |  |

**Strength Session Two**

| Rehab |  | 10min |  |
| --- | --- | --- | --- |
| Exercise | Sets | Reps | Weight/Equipment |
| 4 point kneeling banded hip abduction | 3 | 15 | Band |
| Step up | 4 | 6 | Moderate |
| Single RDL | 5 | 5 | Heavy |
| Squat | 5 | 5 | Heavy |
| Core and mobility |  | 10 min |  |

**Strength Session Three**

| Rehab |  | 10 min |  |
| --- | --- | --- | --- |
| Exercise | Sets | Reps | Weight/Equipment |
| Swiss ball hamstring bridge holds | 3 | 15s | Swiss ball |
| Forearm side plank with hip abduction | 3 | 15 | Body weight |
| Split squat | 5 | 5 | Heavy |
| Leg press | 5 | 5 | Heavy |
| Core and mobility |  | 10 min |  |

**Power Session One**

| Rehab |  | 10 min |  |
| --- | --- | --- | --- |
| Exercise | Sets | Reps | Weight/Equipment |
| 4 point kneeling banded hip abduction | 3 | 15 | Band |
| SL squat | 4 | 6 | Moderate |
| Trapbar deadlift | 4 | 6 | Moderate |
| Skater jumps | 3 | 5/side | Body weight |
| Box jumps | 3 | 5 | Body weight |
| Core and mobility |  | 10 min |  |

**Power Session Two**

| Rehab |  | 10 min |  |
| --- | --- | --- | --- |
| Exercise | Sets | Reps | Weight/Equipment |
| Swiss ball hamstring bridge holds | 3 | 15s | Swiss ball |
| Single RDL | 4 | 4 | Moderate |
| Split squats | 4 | 3 | Moderate |
| Single leg box jumps | 3 | 3 | Body weight |
| Weighted seated vertical jump | 3 | 5 | Light |
| Core and mobility |  | 10 min |  |

Power Session Three

| Rehab |  | 10 min |  |
| --- | --- | --- | --- |
| Exercise | Sets | Reps | Weight/Equipment |
| Bridge with hip external rotation | 3 | 15 | Band |
| Single leg press | 4 | 6 | Moderate |
| Squat | 4 | 6 | Moderate |
| Mountain climber fast | 3 | 5/side | Body weight |
| Jumping lunges | 3 | 5/side | Body weight/light |
| Core and mobility |  | 10 min |  |

| Side plank | Side plank on forearm with ankles stacked |
| --- | --- |
| Forearm plank alternating knee lift | Forearm plank with both knees on the ground. Alternate lifting and lowering one knee at a time, whilst keeping your feet on the ground. |
| Forearm plank with banded step outs | Forearm plank on your feet with a band around your ankles. Alternate stepping one foot out at a time against the band, while keeping your pelvis level |
| Forearm plank with hip rotations | Forearm plank on your feet. Rotate at your hips to alternate dipping one side towards the ground. |
| Dead bug | Lie on your back with your arms in the air and hips flexed to 90 degrees. Alternately lower/raise an opposite arm and leg towards the ground. Focus on deep core. |
| High plank contraleral arm/leg lifts | High plank position. Alternately lift/lower an opposite arm and leg off the ground, whilst maintainig a level pelvis. |
| TA activations with marching | Lying on your back with knees bent and feet on the ground. With your thumbs find your hip bones and then shift slightly in. Staying relaxed and ensuring you are breathing do a small draw in of your belly button. This should make you feel a lift under your thumbs without seeing a big visible core contraction. Maintain this contraction and lift and lower one leg at a time (keeping knee bent) in a marching motion. |
| Russian Twists | Sitting in a V position with knees bent up and feet off the ground, and holding a weight plate in your hands. Rotate L to R with the plate. |
| Plank | High on hands or low on forearms. Focus on deep core engagement and shoulders back and down |
| Leg lowers | Lie on your back, bring both legs up together so they make a 90 degree angle with your body. Slowly lower them down towards the ground. Just before they touch the ground bring them back up to the start position. Focus on using your deep core rather than your hip flexor muscles. |

**Core**

**Mobility**

| Cat Cow | On your hands and knees with wrists directly under your shoulders, and knees directly under your hips. Drop your stomach towards the floor as you lift up your head towards the ceiling, pulling your shoulderblades back and down. Pause and then draw your stomach in and round your back towards the ceiling,  while letting your head relax towards the floor. Pause and repeat slowly changing between the 2 positions. |
| --- | --- |
| Open book stretch | Lie on your side with your head supported on a pillow. Top leg with hip and knee bent to 90 degrees and bottom leg out straight.Reach your arms out in front of you, with your hands together. Rotate the upper arm upwards and outwards from your trunk. Try to rotate as far as you can, without forcing the shoulder. Hold in the maximum position. Note: You can adjust the level at which you rotate by adjusting the angle of your top knee. |
