# Supplementary material for: Case Report: Unilateral resistance and impact loading during knee rehabilitation after grade-2 MCL injury was associated with hip-specific aBMD accrual in an elite female road cyclist
Source: Front Sports Act Living. 2026 Apr 23;8:1823271. doi: 10.3389/fspor.2026.1823271 (PMC13149270; doi:10.3389/fspor.2026.1823271)
Supplement: Supplementary file 3 [file Table2.docx]

| Rehab - VMO, UL |  | 5-10 min |  |
| --- | --- | --- | --- |
| Exercise | Sets | Reps | Weight/Equipment |
| 4 point kneeling banded hip abduction | 3 | 12 | Band |
| Crab walk | 3 | 10 | Band |
| Split stance RDL | 4 | 8 | Light-moderate |
| Squat | 4 | 6 | Moderate-Heavy weight |
| Box jumps | 3 | 5 | Body weight |
| Core and mobility |  | 10 min |  |

**Session One**

| Rehab - VMO, UL |  | 5-10 min |  |
| --- | --- | --- | --- |
| Exercise | Sets | Reps | Weight/Equipment |
| Elevated glute bridge | 3 | 10 | Body-light weight |
| Single leg STS | 3 | 10 | Body weight |
| Step up | 3 | 10 | Body-light weight |
| Dead lift | 4 | 6 | Moderate-Heavy weight |
| Weighted seated vertical jump | 3 | 5 | Light weight |
| Core and mobility |  | 10 min |  |

**Session Two**

| Rehab - VMO, UL |  | 5-10 min |  |
| --- | --- | --- | --- |
| Exercise | Sets | Reps | Weight/Equipment |
| Bridge with hip external rotation | 3 | 15 | Band |
| Clams | 3 | 15 | Band |
| Single leg press | 4 | 8 | Light-moderate weight |
| Squat | 4 | 6 | Moderate-Heavy weight |
| Mountain climber | 3 | 5/side | Body weight/band |
| Core and mobility |  | 10 min |  |

**Session Two**
